# Solute trapping in Al-Cu alloys caused by a 29 Tesla super high static magnetic field

**DOI:** 10.1038/s41598-018-36303-5

**Published:** 2019-01-22

**Authors:** Tianxiang Zheng, Bangfei Zhou, Yunbo Zhong, Jiang Wang, Sansan Shuai, Zhongming Ren, Francois Debray, Eric Beaugnon

**Affiliations:** 1 State Key Laboratory of Advanced Special Steels, Shanghai University, Yanchang Road No. 149, Shanghai, 200072 China; 2 LNCMI, CNRS/UJF/INSA/UPS, 38042 Grenoble, France

## Abstract

Solidification of Al-Cu alloys has been investigated using a 29 Tesla super high static magnetic field (SHSMF). The results show that, by imposing a 29 Tesla SHSMF, the size of primary phases and spacing of eutectic structure have been refined through the increase of undercooling which results from the suppression of diffusion coefficient. The diffusion coefficient of atoms in the liquid matrix decreases to be about 1.2 × 10^−12^ m^2^/s. The lattice constants are reduced and high dislocation density forms in the primary phase, which induces a solute trapping effects. The spacing of (110) plane in Al_2_Cu is corrected to be 4.3123 Å and 4.2628 Å for Al-40 *wt*.%Cu alloys treated without and with a SHSMF. The spacing of (111) plane in Al is corrected to be 2.3351 Å and 2.3258 Å for Al-26 *wt*.%Cu alloys treated without and with a SHSMF. The compression yield strength has been improved by about 42% from 268 MPa to 462 MPa for Al-26 *wt*.%Cu and 42.5% from 248 MPa to 431 MPa for Al-40 *wt*.%Cu. The maximum elastic strain increases from about 2% to 4.3% for Al-26 *wt*.%Cu and from 2% to 4% for Al-40 *wt*.%Cu. It is expected that SHSMF is beneficial to process materials with high mechanical properties.

## Introduction

Al-based metal matrix composites which have been widely applied in area of aerospace and automobile industry for their excellent mechanical properties, lightweight and lower density^1,2^. In these alloys, Al-Cu binary alloy exhibits promising mechanical behavior which is caused by increasing the weight percent of copper in aluminum alloys^3,4^. In addition, by using rapid solidification technique, the mechanical properties can be enhanced due to the solute trapping effect^5^ and grain refinement^6,7^. With the development of high static magnetic field (HSMF) technique, many metal solidification experiments have been successfully carried out under a HSMF which is continuously adjustable in magnetic flux density. Plenty of interesting results such as magnetic orientation^8–10^, grain refinement^11,12^ and thermoelectric magnetic flow^13–16^ *et al*. were revealed under a HSMF (*B* < 20 Tesla). The previous study in reference^14^ mainly focuses on the evolution of the primary Al_2_Cu precipitates in Al-40 *wt*.%Cu hypereutectic alloys under an vertical HSMF (<12 Tesla) by using Bridgman directional solidification method. The effects of HSMF on the eutectic structure have been neglected. And, they did not talk about the orientation induced by the HSMF. It is found that less of studies were done under a super high static magnetic field (SHSMF) (20–30 Tesla). And, especially, the effect of SHSMF on the mechanical property is less reported. Thus, whether new phenomena and theories in such a SHSMF will be found and proposed or not is eager to be known.

Here, we report a new method for solute trapping during the bulk solidification process of Al-26 *wt*.%Cu hypoeutectic alloys and Al-40 *wt*.%Cu hypereutectic alloys under a 29 Tesla super high static magnetic field. The aim of this paper is to investigate the effects of a SHSMF (29 Tesla) on the morphology of primary phases and its compositional distribution by using BSE (2D, backscattered electron, VEGA3 SBH-Easyprobe, TESCAN), TEM (2D, transmission electron microscopy, JEM-2100F, 200 KV), EDS (energy dispersive spectrometer, Hikari XP, EDAX), XRD (12 KW D/max-rC 20–60 KV) and CT (3D, computed tomography, NSI X5000-CT 225). And, the effect of SHSMF on the mechanical property has been discussed.

## Results and Discussion

**Figure 1:**
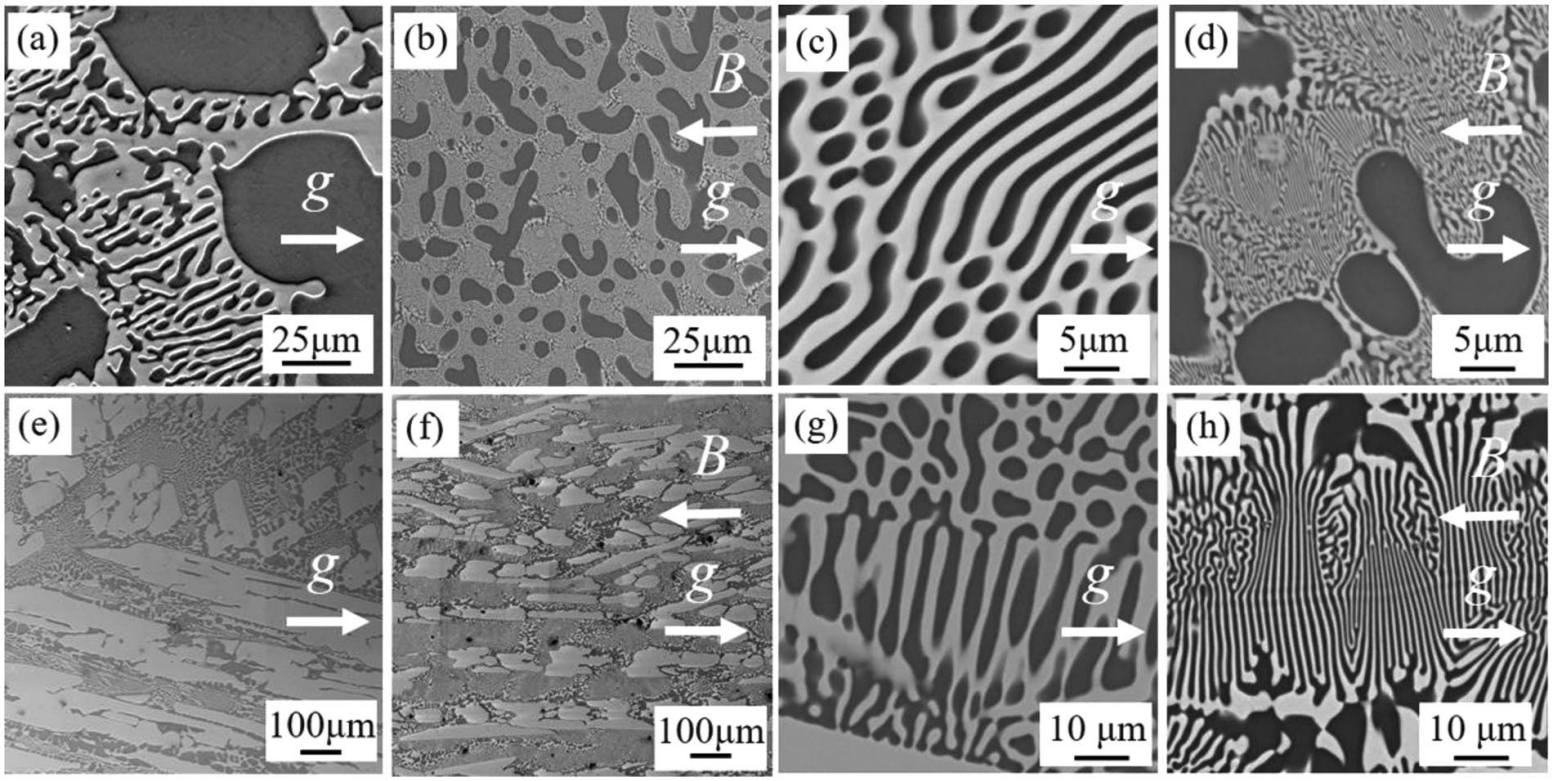
BSE images of Al-Cu alloys. (**a**) 0 T, Al-26 *wt*.% Cu; (**b**) 29 T, Al-26 *wt*.% Cu. (**c**) 0 T, eutectic structure, Al-26 *wt*.% Cu. (**d**) 29 T, eutectic structure, Al-26 *wt*.% Cu. The dark color represents α-Al, the mixed region is eutectic structure. (**e**) 0 T, Al-40 *wt*.% Cu; (**f**) 29 T, Al-40 *wt*.% Cu. (**g**) 0 T, eutectic structure, Al-40 *wt*.% Cu. (**f**) 29 T, eutectic structure, Al-40 *wt*.% Cu. The light color represents Al_2_Cu, the mixed region is eutectic structure. *g* is the gravity.

Figure 1(a,b) are BSE images of Al-26 *wt*.%Cu hypoeutectic alloys solidified under 0 Tesla and 29 Tesla respectively. The dimensions of primary Al dendrites are significantly refined by 29 Tesla SHSMF. Figure 1(c) is the eutectic structure in Al-26 wt.%Cu hypoeutectic alloy treated without SHSMF. And, Fig. 1(d) the corresponding eutectic structure affected by 29 Tesla SHSMF. Compare Fig. 1(c) with (d), the spacing of Al-Al_2_Cu eutectic is refined from about 2–3 μm to tens or hundreds nanometer after applying a 29 Tesla SHSMF. The same phenomena can be found in Al-40 *wt*.%Cu hypereutectic alloys (Fig. 1(e–h)). That is, the dimensions of primary Al_2_Cu phases are significantly refined and the spacing of Al-Al_2_Cu eutectic decreases from about 5–10 μm to about only 1–2 μm. And, it is also found that the normal direction of eutectic lamellar structure is almost parallel to the direction of SHSMF, which can be seen in Fig. 1(f,h). 3D scanning results also show that the primary phase (Al or Al_2_Cu) has been significantly refined, which can be seen in the Supplementary Videos I, II, III and IV. From the Supplementary Video II (Al-26 *wt*.%Cu, 29 Tesla), it is seen that the primary Al dendrites are highly developed and hardly to be detected for its extremely fine size on morphology. A similar situation can be found on Al-40 *wt*.%Cu sample (Supplementary Video IV) which has been treated under a 29 Tesla SHSMF. For Al-40 *wt*.%Cu alloy, based on the computed results, the total volume of Al_2_Cu phase in the scanning part is 21.88 mm^3^. Then, the total volume fraction of Al_2_Cu phase is 48.33%. After the imposition of SHSMF, the total volume of Al_2_Cu phase in the scanning part is 25.71 mm^3^. Then, the total volume fraction of Al_2_Cu phase is 43.14%. It has a decrease (10.74%) in the volume fraction of primary Al_2_Cu precipitates. However, due to the low resolution of CT (2–3 μm in pixel), the volume fraction of primary Al dendrites can not be calculated in Al-26 *wt*.%Cu alloy treated with a 29 Tesla SHSMF. Thus, the calculation of volume fractions of primary Al dendrites in Al-26 *wt*.%Cu alloys (0 Tesla and 29 Tesla) has been canceled.

In liquid-solid phase transformation stage, the refinement of grain is mainly determined by undercooling in the nucleation and growth processes. Based on the previous results, if it coMPares with the thermal energy, no matter the magnetic free energy of Al^17^ and Cu^18^ or other paramagnetism^19^ is negligible. Thus, SHSMF affects the undercooling in this study is attributable to the thermodynamic free energy change caused by the magnetic field on solidification. It is inferred that the undercooling deserves from suppressing the motion of oxide in the melt by applying a SHSMF^18^. That is, the migrating rates of oxides in the melt are thus slowed down. This delays the formation of oxides which is a dynamic stimuli of nucleation and thus increases the undercooling. However, the intrinsic factor, which leads to the formation of oxides and crystal nucleus, is atomic mobility during the solidification process. The atomic diffusion effects should be naturally considered. It can be expressed by viscous flow using the Stokes–Einstein equation derived for the motion of a macroscopic particle in a viscous medium^20,21^. The equation is written as *D* = *k*_B_*T*/6π*η*_eff_*r*, where *D* is the diffusion coefficient, *k*_B_ is the Boltzmann constant and is equal to 1.38 × 10^−23^ J/K, *η*_eff_ is the effective dynamic viscosity and is affected by SHSMF, *r* is an effective hydrodynamic radius of particle and is estimated to be 2.5 Å in the semi-solid state. If we consider the physical properties of Al-26 *wt*.%Cu is similar to Al-30 *wt*.%Cu, the dynamic viscosity (*η*) of liquid Al-26 *wt*.%Cu alloy is estimated to be about 2.2 × 10^−3^ Pa·s at 577 °C (850 K)^22^. In fact, SHSMF affects the Hartman number which is used to calculate the effective dynamic viscosity of liquid matrix. That is *η*_eff_ = *η*·Ha/3 = *B*·*L*·(*ση*)^0.5^/3^23,24^, where Ha is the Hartman number and is calculated to be 2830.4 which greater than 1. In addition, *B* is the magnetic flux density, *σ* is the electrical conductivity of liquid matrix and is estimated to be 3.25 × 10^4^ Ω^−1^·cm^−1^ at 577 °C^22^, *L* is the characteristic length and is 8 mm which is the diameter of the sample). Then, in the semi-solid state (577 °C), the diffusion coefficient of atoms in the liquid matrix is calculated to be 1.13 × 10^−9^ m^2^/s. However, after imposing a 29 T SHSMF, the diffusion coefficient decreases to 1.2 × 10^−12^ m^2^/s. If we consider the interface between a solid grain and its surrounding liquid phase to be a solid-liquid interface, based on constitutional supercooling theory, the critical growth speed of a grain can be described by: *R* = *kG*_*L*_*D*/*m*_*l*_*C*_0_(1-*k*), where *k* is the equilibrium partition coefficient *G*_*L*_ is the temperature gradient in the liquid, *m*_*l*_ is the liquidus slope and *C*_0_ is the initial composition. Previous results indicate that the effect of a HSMF on *k* and *m*_*l*_ is very small^25^. Thus, the size of a grain decreases as the decrease of *D* caused by SHSMF.

**Figure 2:**
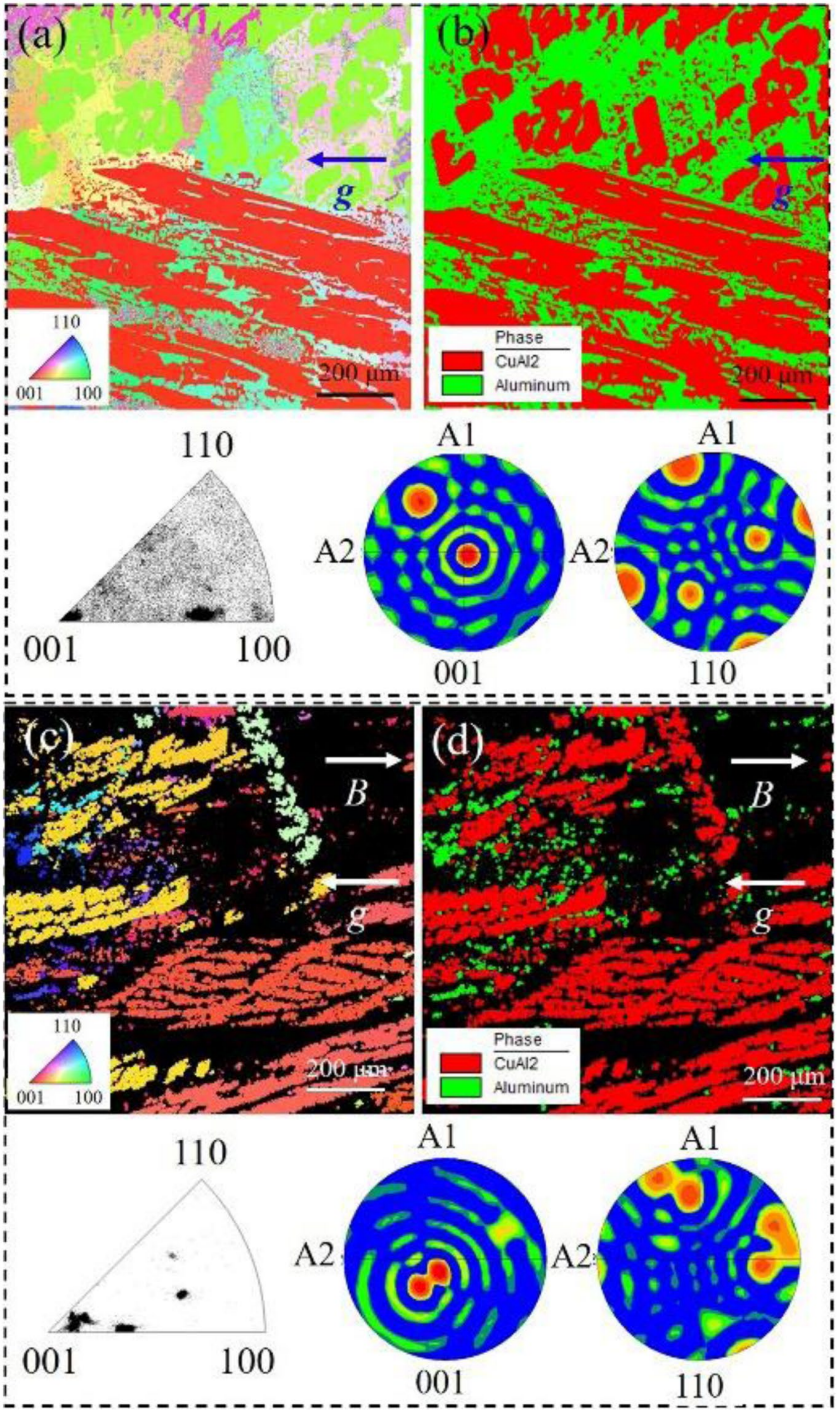
EBSD maps and phase distribution on the longitudinal sections of samples. (**a**) EBSD maps and the corresponding pole and invers pole figures of primary Al_2_Cu phase, 0 T; (**b**) Phase distribution, 0 T; (**c**) EBSD maps and the corresponding pole and invers pole figures of primary Al_2_Cu phase, 29 T; (**d**) Phase distribution, 29 Tesla.

Besides, after the imposition of 29 Tesla SHSMF, the [001] crystal direction of Al_2_Cu precipitations will be oriented to the direction of the SHSMF due to the magnetic anisotropy for Al-40 *wt*.%Cu, which has been extensively reported by previous studies^14,16^. Figure 2 shows the EBSD maps and phase distribution on the longitudinal sections in Al-40 *wt*.%Cu alloy. Examined results in Fig. 2(a,b) show that the primary Al_2_Cu precipitates have two different crystal directions, exhibiting a disordered microstructure. After imposing a vertical 29 Tesla HSMF (Fig. 2(c,d)), the primary Al_2_Cu precipitates were highly oriented in the [11–23 12] and [−6 6 11] crystal directions, which are adjacent to the [001] crystal direction. There is ~13.84° between [11 −23 12] and the [001] crystal direction, and ~38.34° between [−6 6 11] and the [001] crystal direction. CoMPared to the previous results^14,16^, this little angle is attributed to the high cooling rate during the solidification process which lead to less time for the <001> crystal direction of Al_2_Cu precipitates orients to the SHSMF. However, in Al-26 *wt*.%Cu alloy, it is a symmetric structure for Al which is a face centered cubic. Thus, the primary Al phases can not be oriented under the effect of SHSMF.

**Figure 3:**
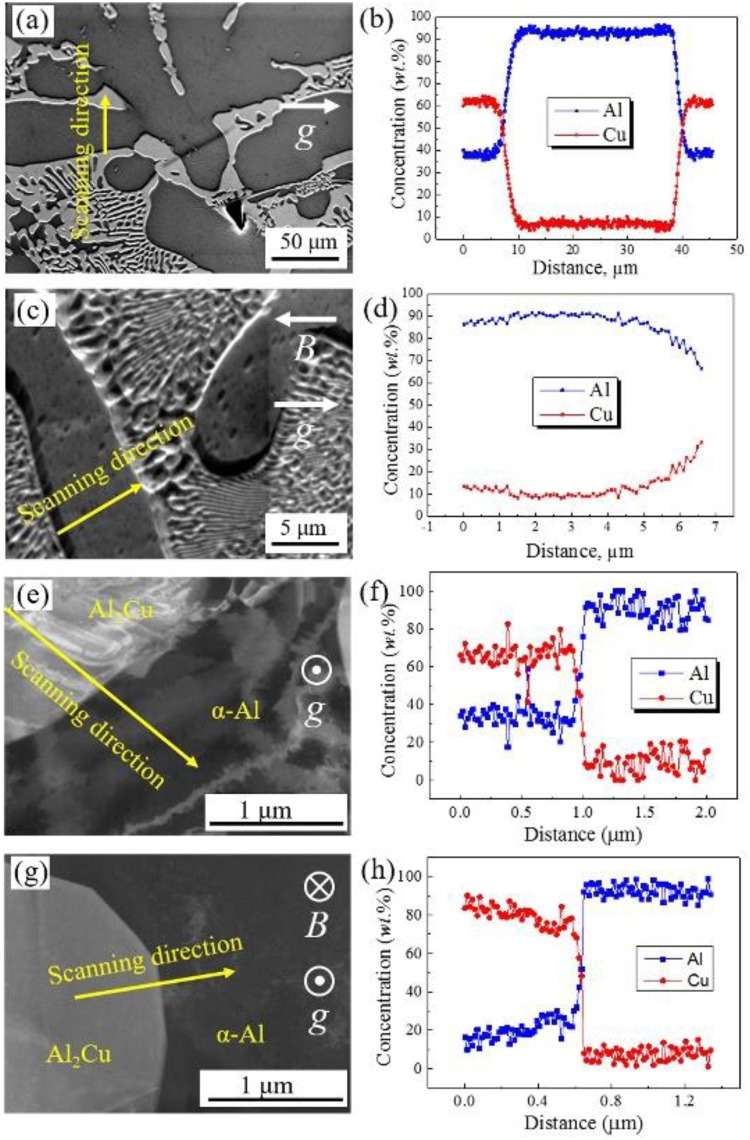
EDS results for Al-Cu alloys treated without and with a SHSMF. (**a**) and (**b**) are SE images and compositional profiles of Al-26 *wt*.%Cu, 0 T; (**c**) and (**d**) are SE images and compositional profiles of Al-26 *wt*.%Cu, 29 T; (**e**) and (**f**) are TEM images and compositional profiles of Al-40 *wt*.%Cu, 0 T; (**g**) and (**h**) are TEM images and compositional profile of Al-40 *wt*.%Cu, 29 T.

Previous studies indicate that the interdiffusion of atoms (Al-Cu^26^, Ni-Al^27^, Mg-Al^28^ and Ni-Ti^29^) were retarded in the solid phases because HSMF had decreased the diffusion constant (*D*_0_) through decreasing the atom vibration frequency and/or the entropy. Compositional distributions of Al and Cu atoms in every Al-Cu samples were detected by EDS (Fig. 3). It is known that the concentration of Cu in primary Al is about 8 *wt*.% in Fig. 3(b). However, the concentration of Cu increased and is a unbalanced distribution around 10 *wt*.% after introducing a 29 Tesla SHSMF. For Al-40 *wt*.%Cu hypereutectic alloys, the compositional profiles of the corresponding region (Fig. 3(e,g)) in nanoscale were examined by TEM which are showed in Fig. 3(f,h). It is easily found that the concentration of Al in primary Al_2_Cu is about 36 *wt*.% in Fig. 3(f). While the concentration of Al decreased and is a unbalanced distribution around 22 *wt*.% for the sample solidified under a 29 Tesla SHSMF (Fig. 3(h)). These results clearly indicate that the SHSMF caused the solutes (Cu atoms) being trapped in the primary solid phases.

**Figure 4:**
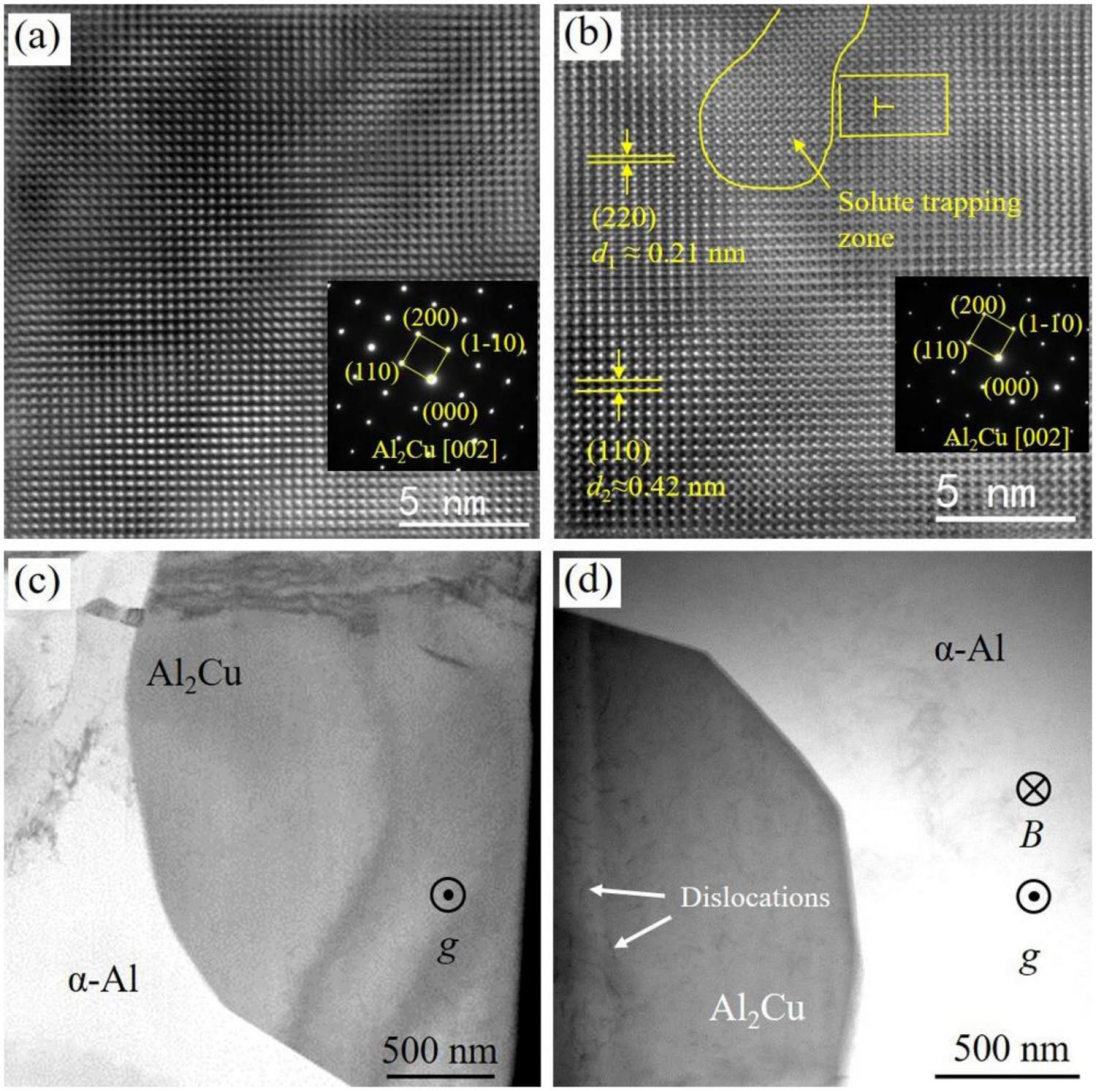
TEM results of Al-40 *wt*.%Cu hypereutectic alloys. (**a**) IFFT and SAED images, 0 T; (**b**) HRTEM and SAED images, 29 T; (**c**) and (**d**) are bright field images under 0 T and 29 T respectively.

**Figure 5:**
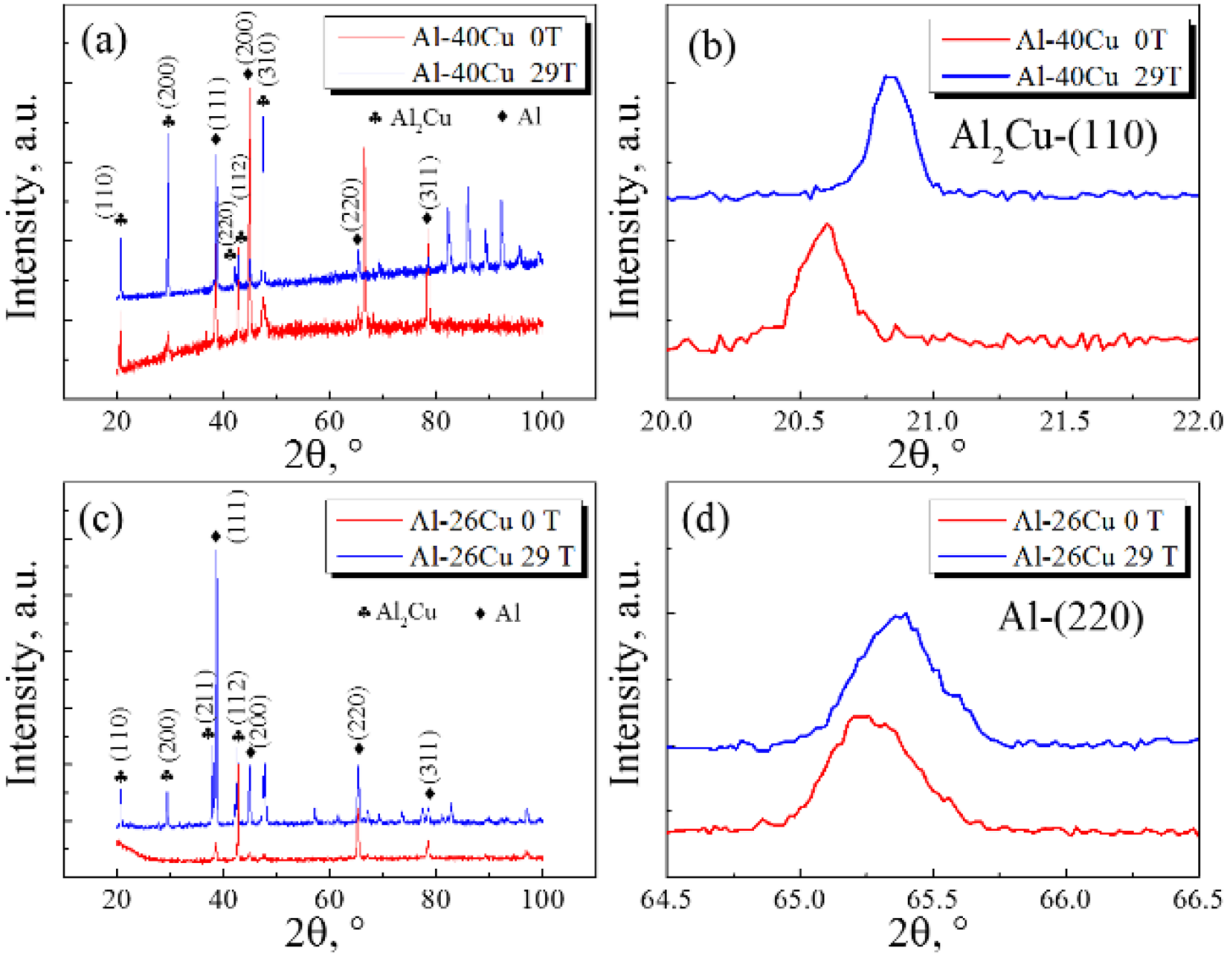
XRD patterns of Al-Cu alloys. (**a**) Full patterns of Al-40 *wt*.%Cu; (**b**) Peak of (110) plane for Al_2_Cu in (a); (**c**) Full patterns of Al-26 *wt*.%Cu; (**d**) Peak of (220) plane for Al in (c).

Figure 4 shows the Inverse Fast Fourier Transform (IFFT), SAED images and bright field images of Al-40 *wt*.%Cu alloys. In Fig. 4(a,b), the arrangement of atoms is regular when the *B* is 0 Tesla while it is irregular when *B* is 29 Tesla. A solute trapping zone is found in the sample treated with 29 Tesla SHSMF. Besides, the spacing of crystal plane at the vicinity of the solute tapping zone has been changed. By using Digital Micrograph software, the new spacing of crystal plane is measured to be about 0.21 nm (*d*_1_) which is (220) crystal plane of Al_2_Cu. This value is right equal to the half of the spacing of (110) crystal plane (*d*_2_) which is measured to be about 0.42 nm. Thus, it is deduced that the formation of the new row of atoms on (220) crystal plane is caused by lattice misfit in the solute trapping zone. As a result, the lattice defects in Al_2_Cu (such as the edge dislocation in Fig. 4(b)) is formed by introducing the SHSMF. In addition, lots of dislocations are found in Al_2_Cu phase when *B* is 29 Tesla SHSMF (Fig. 4(d)). However, Al_2_Cu phase is almost clean when *B* is 0 Tesla (Fig. 4(c)). Based on the results of XRD patterns, 2θ of both the peak of (110) plane in Al_2_Cu and the peak of (220) lattice plane in Al have increased after introducing a 29 T SHSMF (Fig. 5(b,d)). By using Jade 6.0 software, the spacing of (110) plane in Al_2_Cu is corrected to be 4.3123 Å and 4.2628 Å for Al-40 *wt*.%Cu alloy treated without and with a SHSMF. And, the spacing of (111) plane in Al is corrected to be 2.3351 Å and 2.3258 Å for Al-26 *wt*.%Cu alloy treated without and with a SHSMF. Based on Bragg law, both the spacing of (110) plane in Al_2_Cu and the spacing of (111) plane in Al have decreased under the effects of SHSMF. In addition, the corresponding crystallographic cell volumes of Al_2_Cu for Al-40 *wt*.%Cu alloy and Al for Al-26 *wt*.%Cu alloy are respectively calculated to be 179.45901 Å^3^ and 66.16770 Å^3^ when the SHMSF is 0 Tesla. However, when the SHSMF is 29 Tesla, they decrease to 178.98918 Å^3^ (0.26%) and 65.74946 Å^3^ (0.63%) respectively. As we known, the radiuses of Al atom and Cu atom are 1.82 Å and 1.57 Å. And, SHSMF has induced a lattice contraction. Thus, it is deduced that Cu atoms have been trapped in the primary phases during the solidification stage and the concentrations of Cu atoms in both the primary Al_2_Cu and the primary Al have been improved for Al-40 *wt*.%Cu alloy and Al-26 *wt*.%Cu alloy respectively by applying a SHSMF.

Li *et al*.^30^ revealed that lots of dislocations, which was caused by thermal electromagnetic force, existed in primary Al phase during the solidification under a 12 T HSMF. Molotskii and Fleurov^31^ reported that the external magnetic field can depin dislocations from paramagnetic obstacles. This results in an increase in the average dislocations free segment length and, hence, to an increase of dislocation density. So, it is reasonable to deduce that high dislocation density provides more spaces and locations for solutes atoms, which is a solute trapping effect, and thus leads to an unbalanced distribution of atoms in the primary phases and an increase of both yield strength and maximum elastic strain (solute strengthening). Therefore, it is necessary to make an investigation on the mechanical property of these samples.

**Figure 6:**
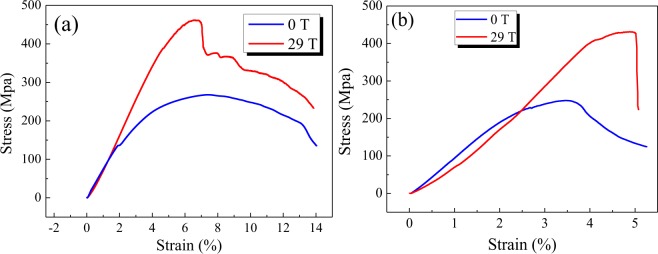
The relationship between the compression stress and the strain tested on and Al-40 *wt*.%Cu alloys solidified without and with a 29 Tesla SHSMF. (**a**) Al-26 *wt*.%Cu alloys; (**b**) Al-40 *wt*.%Cu alloys.

Figure 6 shows a relationship between the compression stress and the strain tested on the Al-26 *wt*.%Cu alloys (Fig. 6(a)) and Al-40 *wt*.%Cu alloys (Fig. 6(b)) solidified without and with a 29 Tesla SHSMF. The absence of yield processes and existence of elastic and fracture stages are found in the curves of all samples. All the curves exhibit almost linear behavior in the early stages which are the elastic stages. After imposing a 29 Tesla HSMF, the compression yield strength has been improved by about 42% from 268 MPa to 462 MPa for Al-26 *wt*.%Cu and 42.5% from 248 MPa to 431 MPa for Al-40 *wt*.%Cu. The maximum elastic strain increases from about 2% to 4.3% for Al-26 *wt*.%Cu and from 2% to 4% for Al-40 *wt*.%Cu. The increase of strength and strain mainly results from the defects in the microstructure, such as grains boundaries (refinement of microstructures) and dislocations inside grains. Based on the Hall-Petch equation, that is *σ* = *σ*_0_ + K*·d*^−0.5^, where *σ* is the yield strength, *σ*_*0*_ is the constant which can be represented by the strength of single crystal, K is the factor and *d* is the size of grains. Thus, with the decrease of grain size, the grains boundaries, strength and strain increase gradually. Besides, as mentioned in the previous section, the orientation of Al_2_Cu precipitates and the normal direction of eutectic lamellar structure being parallel to the direction of SHSMF are also the factors which are in favor of the enhancement of compressive property.

In summary, this letter reports a new method for solute trapping in Al-Cu alloys using a 29 Tesla SHSMF. The size of microstructures, such as primary Al, primary Al_2_Cu and spacing of eutectic, has been significantly refined. SHSMF refined the size of microstructures mainly through the suppression of atom diffusion constant. In addition, the SHSMF induced a lattice contraction and, simultaneously, results in the formation of high dislocation density in the primary phase, which induces a solute trapping effects. For Al-40 *wt*.%Cu alloy, the enhancement of compressive property is attributed to the grains refinement, dislocations in primary Al_2_Cu phases, crystal orientation of primary Al_2_Cu precipitates, solute strengthening, the decrease of the fraction of primary phase and the normal direction of eutectic lamellar structure being parallel to the direction of SHSMF. However, except crystal orientation of primary phase (Al), all other factors will also lead to the enhancement of compressive property for Al-26 *wt*.%Cu alloy.

## Methods

Solidification experiments were performed with Al-26*wt*%Cu hypoeutectic alloys and Al-40*wt*%Cu hypoeutectic alloys prepared using high purity Al (4 N for purity) and Cu (4 N for purity). The alloying process was carried out in a vacuum electromagnetic induction heating furnace under the protection of pure Ar. The sample was heated to about 1000 °C for 20 min to produce an alloy bar with a diameter of 8 mm and a length of about 150 mm through vacuum suction casting. Then, the bar was cut into pieces with 8 mm in diameter and 10 mm in length. Samples were inserted into alumina tubes with an outer diameter of 10 mm and a length of 100 mm and sealed with a graphite tube. The temperature was monitored by a K-type thermocouple which was inserted into a graphite tube. Previous results^32^ show that the high magnetic field has not changed the output voltage of the thermocouple or affected the measuring temperature.

**Figure 7:**
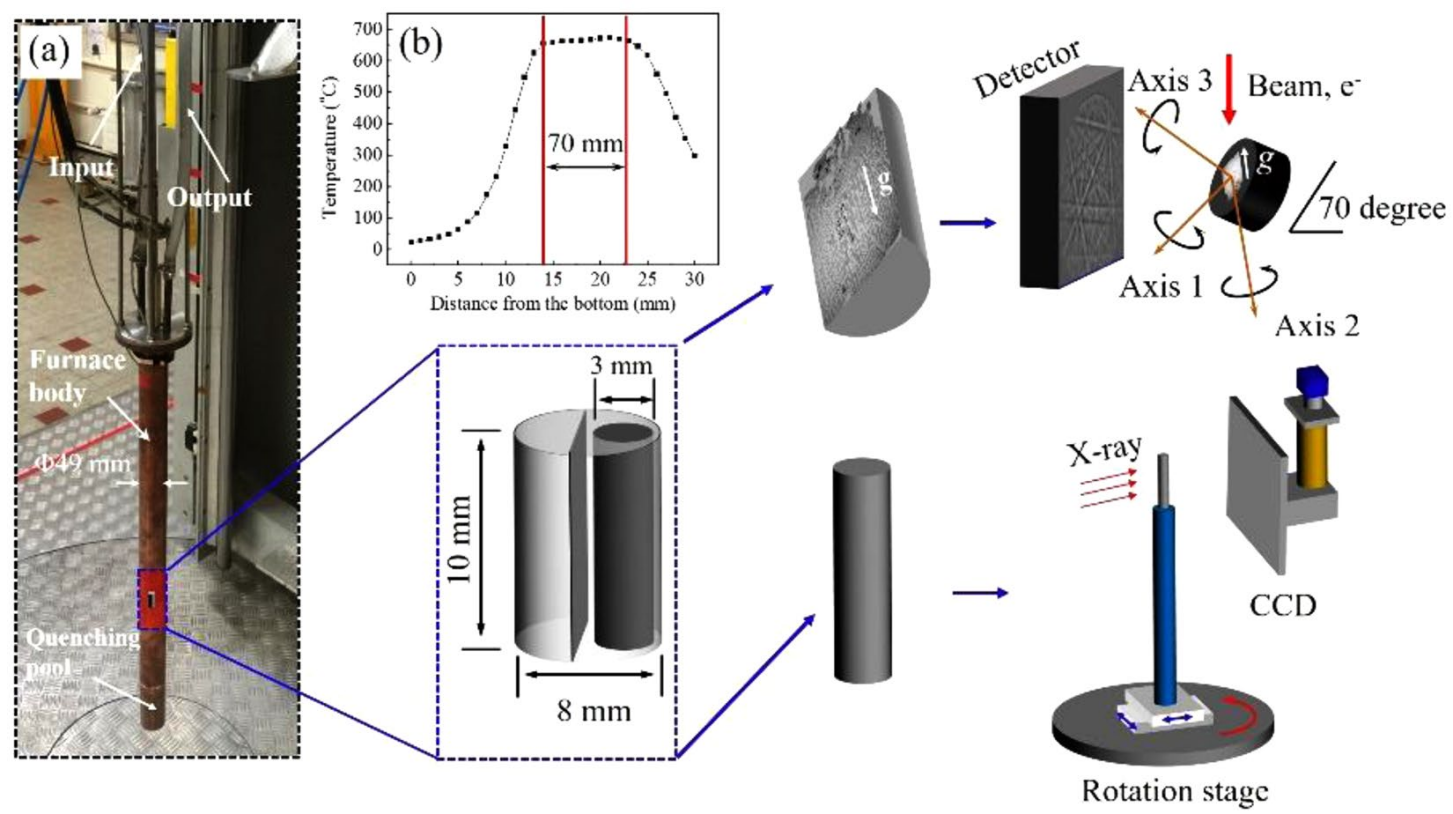
Experimental facilities and sample preparation process of Al-Cu alloys. (**a**) Heating furnace and sample testing processes; (**b**) Temperature distribution profile at 650 °C. *g* is gravitational force.

The sample was placed in the uniform heating region of the electric resistance heating furnace, which was inserted in the bore of the SHSMF facility. SHSMF was produced in a Bitter resistance magnet with a 50 mm room temperature bore at LNCMI (Grenoble, France). The temperature was 700 °C, which was held for 1 h. After the holding time, the melt was cooled to ambient temperature at a cooling rate (*R*) of 50 °C/min with or without a vertical SHSMF. SHSMF was switched on after 30 min of holding time and switched off when the sample was cooled to ambient temperature. To properly fit the bore 50 mm in diameter, a special electric resistance furnace (Fig. 7(a)) was designed. The furnace was made up of a copper tube 49 mm in diameter and a molybdenum resistance wire 1 mm in diameter. In addition, a water cooling system was designed around the furnace. The temperature distribution in the furnace is shown in Fig. 7(b). The constant temperature zone is about 70 mm in length. Figure 7(a) also describe two testing processes of ingot after the solidification experiment. Every solidified sample was cut along the central axis as two symmetrical parts. One of the parts was polished mechanically, followed by FIB (FEI, 600i) for TEM tests and ion milling (IM 4000, Hitachi) for EBSD (Hikari XP, EDAX) tests (the upper process in Fig. 7(a)). The other part was cut as a cylinder (Φ 3 × 8 mm) for a 3D computed tomography scanning test (2–3 μm in pixel), which can be seen in other process of Fig. 7(a). After the 3D computed tomography scanning test, a cylinder specimen with a dimension of Φ 3 × 5 mm was cut from the previous cylinder for the compressive tests. The mechanical properties (compression test) was performed with a strain rate of 5 × 10^−3^ mm/s in air at ambient temperature.

## Electronic supplementary material

Supplementary Video 1

Supplementary Video 2

Supplementary Video 3

Supplementary Video 4
